# The overlap syndrome of urticaria and gastroesophageal reflux disease

**DOI:** 10.1371/journal.pone.0207602

**Published:** 2018-11-20

**Authors:** Ernesto Aitella, Fabio De Bartolomeis, Alfonso Savoia, Massimo Fabiani, Marco Romano, Corrado Astarita

**Affiliations:** 1 Aggregate Post Graduate School in Allergology and Clinical Immunology of Campania University L. Vanvitelli, Naples, Italy and of Federico II University, Naples, Italy; 2 Department of Precision Medicine, Division of Internal Medicine, Section of Allergology and Clinical Immunology, Campania University L. Vanvitelli, Naples, Italy; 3 Post Graduate School in Allergology and Clinical Immunology of Campania University L. Vanvitelli, Naples, Italy; 4 Infectious Diseases Department, Italian National Institute of Health (ISS), Rome, Italy; 5 Department of Precision Medicine, Division of Gastroenterology, Campania University L. Vanvitelli, Naples, Italy; Federal University of Sergipe, BRAZIL

## Abstract

**Background:**

One-quarter of systemic symptoms associated with chronic spontaneous urticaria (CSU) are related to gastrointestinal complaints (GICs).

**Objectives:**

To investigate the prevalence and features of urticaria-overlapping GICs.

**Methods:**

In this retrospective cross-sectional survey, 1426 consecutive outpatients were observed at our University Department. Only patients suffering from urticaria or GICs with a complete diagnostic work-up including serum total IgE level (Tot-IgE), differential blood count and urticaria activity score (UAS), were evaluated.

**Results:**

Among different GICs, gastroesophageal reflux disease (GERD) was the most frequent syndrome observed (15.4%; 95%CI: 13.6–17.3). The prevalence of overlap syndrome for urticaria and GERD was 5.9% (95%CI: 4.7–7.2). In urticaria-patients, the prevalence of GERD was four-fold higher than in patients without hives (44% vs. 11%, p<0.001). UAS was significantly higher in urticaria and GERD overlap syndromes vs. isolated urticarias. In patients with GERD or acute/chronic urticaria or overlap syndrome, Tot-IgE and eosinophil blood count (EBC) differed significantly, with a stepwise increase in their values; from the subgroup of patients with GERD only, to that with overlap of CSU to GERD. Prevalence values for urticaria overlapping with GERD were three- and two-fold higher in CSU and in long-duration GERD cases respectively compared to acute urticaria or short-duration GERD cases. Similar to Th2 pathology models, CSU and GERD overlap syndrome was significantly and independently associated with Total-IgE ≥100IU/ml or EBC ≥250/mmc compared to CSU or GERD. Endoscopic/bioptic findings of non-erosive reflux disease (NERD) or Barrett’s esophagus (BE) were more frequent in chronic overlap syndrome than in GERD-patients.

**Conclusions:**

GERD was the most frequent GIC in patients with urticaria. Overlap syndrome was more frequent among patients with CSU, where this syndrome was associated with higher values of UAS, Tot-IgE, EBC and frequencies of NERD and BE. These results suggest that overlap syndrome is frequently a chronic syndrome with a Th2-like profile.

## Introduction

Urticaria is a common and heterogeneous skin disorder characterised by a mast cell-driven vascular reaction causing wheals, itch and /or angioedema in response to several either identifiable infectious, allergic, physical, chemical and psychological stimuli or other unidentifiable stimuli [1–6] Due to known or unknown causes and excluding physical stimuli, when recurrence of symptoms persist for ≤6 or >6 weeks, this urticarial skin eruption is defined as acute spontaneous urticaria (ASU) or chronic spontaneous urticaria (CSU), respectively [1]. Although the pathophysiology of CSU is not yet completely understood, a strong characteristic of the disease is the specific increased activation and/or degranulation of skin mast cells (MCs). These are primed to increase the release of proinflammatory and vasoactive mediators through different immunological or non-immunological stimuli and synergistic/sequential different pathomechanisms [6–12]. Over the past five years, clinical studies have demonstrated the efficacy and safety of the monoclonal antibody omalizumab in the treatment of spontaneous and inducible chronic urticaria [13–15]. However, beyond its’ ability to bind circulating IgE, the underlying mechanisms of action are not yet fully understood. [16].

Because human skin MCs are stabilised by anti-IgE agents [17], omalizumab may decrease the degranulation/activation threshold of skin MCs by non-immunological stimuli such as Substance P (SP) [18–20]. Of note, SP activates human skin MCs predominantly via Mas-related G-protein coupled receptor-X2 (MRGPRX2), that is normally expressed on the skin MC subset rich in tryptase and chymase (MC_TC_) in humans [21]. Furthermore, MRGPRX2 has also been observed to be over-expressed on skin MCs of patients suffering from CSU, the mechanisms of which are still unknown [22, 23]. In addition, SP activates human eosinophils [24] that, due to their accumulation in CSU lesions [25] and ability to induce a further degranulation of MCs [22], may play an important role in the pathophysiology of CSU [26]. With regard to prevalence data from clinical studies, in a recent clinical survey involving patients affected with CSU, 64% of patients reported concomitant systemic complaints, consisting of gastrointestinal complaints (GICs) in more than a quarter of cases. Gastrointestinal complaints in these patients were also associated with greater disease burden and higher serum tryptase levels [27]. Preliminary observations from our group suggest a high prevalence of symptoms and endoscopic signs of gastro-esophageal reflux disease (GERD) among Italian CSU patients with contemporary GICs [28]. Moreover, GERD is recognised as a syndrome that is prevalent in Southern Italy [29] and shares features with CSU such as elevated plasma SP levels [20, 30, 31], which may have an important role in pathogenesis and clinical severity of hives [20]. On this basis, we have conducted a retrospective cross-sectional survey to estimate the prevalence of urticaria and GERD overlap syndrome in patients affected with urticaria and/or GICs and to investigate its associated demographic, clinical and biological features.

## Methods

### Study population and data source

We examined all patients affected by urticaria and/or GICs among a sample of 1426 newly observed consecutive patients who attended the Allergy and Clinical Immunology Section of our University Multispecialty Department from October 2013 to December 2014.

Diagnosis of urticaria and/or GICs was confirmed by a clinical allergist/immunologist or a gastroenterologist based at the Department where the study was conducted. Among GICs, GERD was suspected on the basis of a weekly/daily recurrence of heartburn and/or reflux [32] and confirmed in almost all cases by endoscopy. Patients with suspect physical urticaria, intestinal or extra intestinal parasitosis, infections other than Helicobacter pylori (HP) and drug-induced urticaria were excluded from the study. Data were retrospectively retrieved from patients’ electronic registry and rendered anonymous for analysis by omitting any possible personal identifiers. The Ethics Committee of University-Hospital of Campania University “Luigi Vanvitelli”, Naples, Italy approved the study.

### Endoscopic examination data

Although missing in all ASU cases, results of gastrointestinal endoscopic examination were available for all patients with clinically predictable GERD and used to confirm and grade it [33]. Diagnosis of non-erosive reflux disease (NERD) resulted from the endoscopic absence of mucosal breaks associated with a marked improvement in heartburn and/or acid reflux by a PPI test [34, 35]. Diagnostic endoscopy for suspect Barrett’s esophagus (BE) was always investigated by histological examination [36, 37].

### Diagnostic work-up

In addition to physical examination, basic demographic data and current Urticaria Activity Score (UAS) during clinical observation, all patients completed our relatively extensive diagnostic work-up, to avoid any potential diagnostic bias. Work-up included a complete blood count with differential count and main liver function tests, the determination of erythrocyte sedimentation rate, C-reactive protein levels, urine routine examination, stool examination for parasites, dosage of total IgE level (ImmunoCap Total IgE-Phadia AB-Uppsala, Sweden) and the presence/absence of infection by HP as determined by 13C-Urea breath/HP, stool antigen test or endoscopic biopsy. Our data of interest were demographic data, eosinophil blood count (EBC), total IgE serum level (Tot-IgE), the presence/absence of H. Pylori and current UAS.

### Urticaria activity score

All patients with urticaria we observed were receiving treatment with different dosages of different non-sedating antihistamines and they consented to the temporary suspension of drugs during the week of urticaria activity determination. For this purpose, patients used the simple and validated UAS that assesses daily pruritus and number of hives; each component of the UAS was scored on a scale of 0 to 3; the 2 scores are added together for a daily total of 0 to 6 which were added up (sum of each day) over a week, providing the UAS7 [38].

### Statistical analysis

Demographic, clinical and main biological data of patients were presented as absolute frequencies with percentage for categorical variables or median values with interquartile range for continuous variables. Differences among sub-groups of patients were evaluated by bivariate analysis using the Chi-squared test for categorical variables, and the non-parametric Wilcoxon rank sum test or Kruskall-Wallis test respectively, for comparison of continuous variables between two and three or more sub-groups. Some continuous variables (age, Tot-IgE and EBC) were also analysed after recoding into categories of sufficient size to allow for stratification. The results of the Chi-squared test performed for differences among sub-groups after recoding were completely consistent with those derived from the non-parametric tests run for continuous variables before categorization.

We calculated the prevalence of urticaria, GICs and their overlap syndromes in the whole sample and by sub-groups of patients also considering their 95% confidence interval (CI). The association between syndrome of urticaria overlapping GERD with urticaria duration (chronic vs. acute) and between overlap syndrome and GERD duration (> 12 months vs. ≤ 12 months) was evaluated using a multivariate log-binomial model which was controlled for potential confounding effect due to sex, age and presence of HP. The same model was used to evaluate the independent association of chronic urticaria and GERD overlap syndrome with Tot-IgE, EBC and their interaction in both CSU and GERD patients. The strength of associations were described by the adjusted prevalence ratios (PR) and their 95% CI. All p-values less than 0.05 were considered statistically significant. All analysis was performed using Stata/MP version 1.3 (StataCorp LP, Texas, USA).

## Results

### Prevalence of GICs and spontaneous urticaria

From the entire sample (n = 1426), 290 patients reported GICs (20.3%; 95% CI, 18.2–22.4). A total of 191 patients were suffering from urticaria (13.4%; 95% CI, 11.7–15.7) of which 71 were from ASU (5.0%; 95% CI, 3.9–6.2) and 120 from CSU (8.4%; 95% CI, 7.0–10.0). Among GICS, GERD was the most frequent syndrome (15.4%; 95% CI, 13.6–17.3), followed by irritable bowel syndrome (IBS) (12.2%; 95% CI, 10.2–17.3) and dyspepsia (11.2%; 95% CI, 9.6-12-9). However, overlap with IBS and/or dyspepsia was present in 150 (10.6%; 95% CI, 9.0–12.2) patients suffering from GERD. The overlap of urticaria with GERD was observed in 84 patients (5.9%; 95% CI, 4.7-7-2) while overlap of urticaria with other GICs was slightly less frequent (Table 1).

**Table 1:** Prevalence of urticaria and main gastrointestinal complaints in all 1426 consecutive patients.

	Overall prevalence *N*. (%; 95% CI)
• **URTICARIA**	• **191 (13.4; 11.7–15.3)**
✓ *Acute*	✓ *71 (5*.*0; 3*.*9–6*.*2)*
✓ *Chronic*	✓ *120 (8*.*4; 7*.*0–10*.*0)*
• **GICs**^**^**^	• **290 (20.3; 18.2–22.4)**
✓ *GERD*^***^^***^	✓ *220 (15*.*4; 13*.*6–17*.*3)*
✓ *IBS*^***^^^***^	✓ *174 (12*.*2; 10*.*2–14*.*0)*
✓ *DYSPEPSIA*	✓ *159 (11*.*2; 9*.*6–12*.*9)*
✓ *GERD plus IBS and/or DYSPEPSIA*	✓ *150 (10*.*6; 9*.*0–12*.*2)*
• **URTICARIA and GERD**	• **84 (5.9; 4.7–7.2)**
• **URTICARIA and IBS**	• **63 (4.4; 3.4–5.6)**
• **URTICARIA and DYSPEPSIA**	• **51 (3.6; 2.7–4.7)**

**^GICs** = Gastrointestinal complaints

**^^GERD** = Gastroesophageal reflux disease

**^^^IBS** = Irritable bowel syndrome.

The results shown in Table 2 revealed a significantly higher frequency of GERD, IBS and dyspepsia in patients suffering from urticaria compared to those who were not (p<0.001). GERD presence as well as its association with urticaria (p<0.001) was confirmed through esophagogastroduodenoscopy in all clinically–diagnosed patients who consented to the endoscopic examination. It is worth highlighting that while the frequency of celiac disease was similar between the two groups, chronic inflammatory bowel diseases were absent among patients with urticaria but over-represented (0.8%) in patients without hives, although this difference did not attain statistical significance.

**Table 2:** Prevalence of gastrointestinal complaints by the presence or absence of urticaria in all 1426 consecutive patients.

	URTICARIA CASES		NON URTICARIA CASES		
	*N*. = 191		*N*. = 1235		
	*n*.	%	*n*.	%	p-value
**# Patients positive for any GICs**^**+**^	**92**	**48.2**	**198**	**16.0**	**<0.001**
**# Patients positive for each single GIC**					
• **GERD**^**++**^ **–** *	**84**	**44.0**	**136**	**11.0**	**<0.001**
• **GERD**^**++**^ **–** **^**	**70**	**36.6**	**136**	**11.0**	**<0.001**
• **IBS**^**§**^ **–** */^	**63**	**33.0**	**111**	**9.0**	**<0.001**
• **DYSPEPSIA–** */^	**51**	**26.7**	**108**	**8.7**	**<0.001**
• **IBD**^**§§**^ **–** **^**	**0**	**0.0**	**10**	**0.8**	**0.212**
• **CELIAC DISEASE–** **^**	**1**	**0.5**	**6**	**0.5**	**0.945**
• **GALLSTONE –^**	**0**	**0.0**	**1**	**0.2**	**0.694**
• **OTHERS GICs–** **^**	**0**	**0.0**	**2**	**0.2**	**0.578**

^**+**^**GICs** = Gastrointestinal complaints

^**++**^**GERD** = Gastroesophageal reflux disease^;^

^**§**^**IBS** = Irritable bowel syndrome

^**§§**^**IBD** = inflammatory bowel diseases

* = Clinical diagnoses

**^** = endoscopic diagnosis or diagnoses confirmed by other instrumental or laboratory investigations.

### Demographic data, clinical and main biological features of patients suffering from GERD and/or urticaria

Table 3 summarises the main features of patients suffering from urticaria and/or GERD by their clinical presentation. In this subgroup of 327 patients, approximately one-quarter (n = 84) suffered from ASU (n = 14, 4.3%) or CSU (n = 70, 21.4%) that were overlapping with a pre-existing GERD, while the remaining 243 patients were exclusively suffering from GERD (n = 136, 41.6%), ASU (n = 57, 17.4%) or CSU (n = 50, 15.3%). Distribution of sex, age and HP co-infection did not differ among the five groups. Urticaria and GERD overlap syndrome was significantly more frequent in patients suffering from CSU compared to those from ASU (p<0.001). The duration of GERD related symptoms before onset of urticaria was significantly longer in patients suffering from subsequent overlap of urticaria compared to patients with isolated GERD (p<0.001) as well as in GERD cases with overlap of CSU than in those with ASU (p<0.001). As measured by UAS7, patients suffering from ASU or CSU overlap with GERD were observed to have a greater severity of hive recurrence compared to those suffering only from isolated ASU (p<0.001) or CSU (p<0.001), respectively. A relative longer duration of urticaria in patients with overlap of ASU with GERD compared to patients suffering from isolated ASU (p<0.001) was also observed.

**Table 3:** Demographic and clinical features of patients suffering from gastroesophageal reflux disease (GERD) and/or urticaria according to different clinical presentations.

	CLINICAL PRESENTATIONS						
	a	b	c	d	e		
	GERD	ASU°	Acute UGOS*	CSU°° #	Chronic UGOS*	*Total*	*p-value*
	(n. = 136)	(n. = 57)	(n. = 14)	(n. = 50)	(n. = 70)	*(N*. *= 327)*	
	n. (%)”	n. (%)”	n. (%)”	n. (%) “	n. (%)”	n. (%)”	
**Sex**							**@**
• **Male**	**54 (39.7)**	**25 (43.8)**	**8 (57.1))**	**18 (36.0)**	**21 (30.0)**	**127 (38.8)**	**All: 0.185**
• **Female**	**82 (61.3)**	**32 (56.2**	**6 (42.9**	**32 (64.0)**	**49 (70.0)**	**200 (61.2)**	
**Age class (years)**							**@**
• **<30**	**28 (20.6)**	**14 (24.6)**	**3 (21.4)**	**7 (14.0)**	**16 (22.9)**	**66 (20.2)**	**All: > 0.05**
• **30–39**	**31 (22.8)**	**22 (38.6)**	**5 (35.7)**	**18 (36.0)**	**23 (32.9)**	**99 (30.3)**	
• **40–49**	**47 (34.5)**	**12 (21.0)**	**3 (21.4)**	**17 (34.0)**	**19 (27.1)**	**100 (30.6)**	
• **≥50**	**30 (22.1)**	**9 (15.8)**	**3 (21.4)**	**8 (16.0)**	**12 (17.1)**	**62 (18.9)**	
**Helicobacter pylori coinfection**							**@**
• **Yes**	**46 (33.8)**	**24 (42.1)**	**5 (35.7)**	**16 (32.0)**	**28 (40.0)**	**119 (36.4)**	**All: = 0.735**
• **No**	**90 (66.2)**	**33 (57.9)**	**9 (64.3)**	**34 (68.0)**	**42 (60.0)**	**208 (63.6)**	
**UGOS presence**						***(N*. *= 191)***	**@**
• **Yes**	**NA**	**14 (19.7)**		**70 (58.3)**		**84 (44.0)**	**<0.001**
• **No**		**57 (80.3)**		**50 (41.7)**		**107 (56.0)**	
**GERD duration (months)^**						***(N*. *= 220)***	**§**
**– Median [IQR]**	**10 [8–12]**	**NA**	**9 [1–12]**	**NA**	**13.5 [10–18]**	**12 [8–14]**	**All:<0.001**
							**e > a: <0.001**
							**e > c: = 0.002**
**Urticaria duration (weeks)**						***(N*. *= 191)***	**§**
**– Median [IQR]**	**NA**	**1.5 [1.5–2]**	**3 [3–4]**	**17.5 [14–22]**	**16 [13–20]**	**12 [2–19]**	**All: < 0.001**
							**c > b: <0.001**
							**d vs. e: = N**
**UAS7**						***(N*. *= 191)***	**§**
**– Median [IQR]**	**NA**	**19 [18–21]**	**27 [24–28]**	**14.3 [13–19]**	**22 [20–26]**	**20 [16–23]**	**All: < 0.001**
							**c > b: < 0.001**
							**e > d: < 0.001**

**“**= continuous variables are presented as median and IQR

**^** = Duration of GERD before urticaria appearance

**°ASU** = Acute Spontaneous Urticaria

**°° CSU** = Chronic Spontaneous Urticaria

*Acute/chronic UGOS = Acute/chronic Urticaria and GERD Overlap Syndrome

**@ =** Chi-square test

**§** = Wilcoxon rank sum test (comparison between two groups) or Kruskall-Wallis test (comparison among three or more groups)

**IQR** = Interquartile range

**UAS7** = Urticaria activity score over a week

**NA** = Not applicable.

**NS** = Not significant.

**Fig 1:**
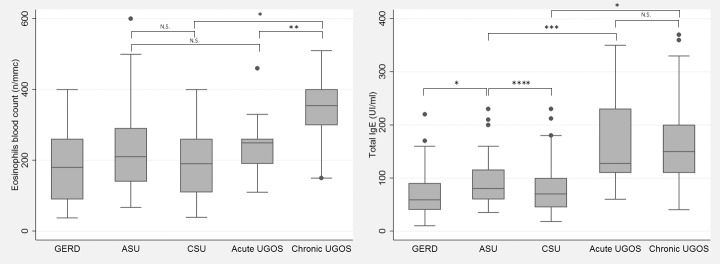
Blood eosinophils and total IgE levels, according to different clinical presentations of gastroesophageal reflux disease (GERD) and/or urticaria. Eosinophils and total IgE are significantly more elevated in all cases of urticaria and GERD overlap syndrome (UGOS) compared to isolated cases of GERD or urticaria. To be noted in the figure, where some of the significant differences are highlighted, how both values show a stepwise increase from the isolated GERD group up to the chronic urticaria and GERD overlap syndrome (Chronic UGOS) group. *: p <0.00001; **: p = 0.0018; ***: p = 0.003; ****: p = 0.028; **N.S. =** Not significant.

Levels of serum total IgE and EBC in patients affected from non-associated diseases (GERD, ASU and CSU) or urticaria and GERD overlap syndromes are presented in Fig 1 and Table 4. Tot-IgE levels and EBCs significantly differed among the groups (p <0.001), with lower values observed in patients suffering from GERD, and higher values in those suffering from chronic urticaria and GERD overlap syndrome. With regard to non-associated diseases, while total IgE levels were similar in GERD and CSU, they markedly increased in ASU compared to GERD (p <0.00001) and moderately when compared to CSU (p = 0.027), while EBCs remained similar across groups. Considering the overlap syndromes between GERD and ASU/CSU, while levels of total IgE were similar in both syndromes, EBC was significantly higher in overlapping syndrome of CSU with GERD than in that of ASU with GERD. Compared to isolated GERD, total IgE level increased significantly in both overlap syndromes, but the increase was higher in patients with overlapping CSU (z-score = -8.7) than those with ASU (z-score = -4.4). Similarly, EBC also increased significantly but not to a similar extent in overlap syndromes of ASU (p = 0.0348) or CSU (p<0.00001) with GERD. Compared to the corresponding isolated urticaria, total IgE increase was statistically significant in both overlap syndromes, but the increase between isolated ASU and ASU overlap with GERD was lower (p = 0.00328) than that observed between isolated CSU and CSU overlap with GERD (p<0.00001). In contrast, EBC only increased in CSU and GERD overlap syndrome (p<0.00001).

**Table 4:** Total IgE and Eosinophils blood count analysed by different clinical presentations and after recoding into categories of approximately equivalent size patients suffering from gastroesophageal reflux disease (GERD) and/or urticaria.

	CLINICAL PRESENTATION						
	a	b	c	d	e		
	GERD	ASU°	CSU°°	Acute	Chronic	*Total*	
				UGOS*	UGOS*		*p*
	n. = 136	n. = 57	n. = 50	n. = 14	n. = 70	*N*. *= 327*	*(Chi-square-test)*
	n. (%)	n. (%)	n. (%)	n. (%)	n. (%)	N. (%)	
**Total IgE (IU/ml)**							
• **< 50**	**50 (36.8)**	**4 (7.0)**	**13 (26.0)**	**0 (0.0)**	**1 (1.4)**	**68 (20.8)**	- a vs b: 0.00027a vs c: NS
							-a vs c: NS
• **50–79**	**45 (33.1)**	**23 (40.4**	**19 (38.0)**	**3.(21.4)**	**8.(11.4)**	**98 (30.0)**	- a vs d: 00002
							- a vs e: <0.00001
• **80–149**	**33 (24.2)**	**24 (42.1)**	**13 (26.0)**	**5 (35.7)**	**24 (34.3)**	**99 (30.3)**	- b vs c: 0.044
							- b vs d: 0.027
• **≥ 150**	**8 (5.9)**	**6.(10.5)**	**5 (10.0)**	**6 (42.9)**	**37 (52.9)**	**62 (18.9)**	- c vs e: <0.001
							- d vs e: NS
**Eosinophils blood count (n./mmc)**							
• **<120**	**41 (30.1)**	**12 (21.0)**	**13 (26.0)**	**2 (14.3)**	**0 (0.0)**	**68 (20.8))**	- a vs b: NS
							- a vs c: NS
• **120–229**	**47 (34.6)**	**18 (31.6)**	**16 (38.0)**	**2 (14.3)**	**7 (10.0)**	**90 (27.5)**	- a vs d: NS
							- a vs e: <0.00001
• **230–329**	**39 (28.7**	**18 (31.6)**	**14 (26.0)**	**8 (57.1)**	**15 (21.4)**	**94 (28.8)**	- b vs c: NS
							- b vs d: NS
• **≥ 330**	**9 (6.6)**	**9 (15.8)**	**7 (14.0)**	**2 (14.3)**	**48 (68.6)**	**75 (22.9)**	- c vs e: <0.00001
							- d vs e: 0.0007

**°ASU** = Acute Spontaneous Urticaria

**°° CSU** = Chronic Spontaneous Urticaria

*Acute/chronic UGOS = Acute/chronic Urticaria and GERD Overlap Syndrome.

**NS** = Not significant.

After adjusting for sex, age, and HP co-infection, the prevalence of urticaria and GERD overlap syndrome was three-fold higher in CSU compared to ASU patients (adjusted PR = 2.99; 95% CI, 1.82–4.92), while overlap syndrome-prevalence was two-fold higher in long-duration GERD compared to short-duration cases of GERD (adjusted PR = 2.06; 95% CI, 1.49–2.83) (Table 5).

**Table 5:** Factors independently associated with urticaria and GERD overlap syndrome (UGOS) compared to patients suffering from isolated urticaria [A] or isolated gastroesophageal reflux disease (GERD) [B].

	CLINICAL PRESENTATION: UGOS [A]				CLINICAL PRESENTATION: UGOS [B]			
	ISOLATE ASU+CSU N = 107	UGOS N = 84			ISOLATE GERD N = 136	UGOS N = 84		
	*n (%)*	*n (%)*	*Adjusted PR°* *(CI 95%)*	p-value*	*n (%)*	*n (%)*	*Adjusted PR°* *(CI 95%)*	p-value*
**Sex**								
Male	45 (60.8)	29 (39.2)	1		53 (64.6)	29 (35.4)	1	
Female	62 (52.0)	55 (47.0)	1.06 (0.77–1.45)	0.739	83 (60.1)	55 (39.9)	0.96 (0.68–1.36)	0.833
**Age classes (years)**								
< 30	20 (51.3)	19 (48.7)	1		27 (58.7)	19 (41.3)	1	
30–39	40 (58.8)	28 (41.2)	0.84 (0.57–1.22)	0.356	31 (52.5)	28 (47.5)	1.11 (0.75–1.65)	0.594
40–49	30 (56.9)	22 (43.1)	0.80 (0.54–1.19)	0.274	48 (68.6)	22 (31.4)	0.75 (0.48–1.18)	0.210
≥ 50	17 (53.1)	15 (46.9)	0.94 (0.62–1.44)	0.786	30 (66.3)	15 (33.3)	0.82 (0.50–1.34)	0.434
**HP–Infection**								
Negative	67 (56.8)	51 (43.2)	1		90 (63.8)	51 (36.2)	1	
Positive	40 (54.8)	33 (44.2)	1.11 (0.83–1.48)	0.471	46 (58.2)	33 (41.8)	1.16 (0.86–1.18)	
**Urticaria duration**								
≤ 6 weeks (ASU)	57 (80.3)	14 (19.7)	1		NA	NA	NA	NA
> 6 weeks (CSU)	50 (41.6)	70 (58.4)	2.99 (1.82–4.92)	< 0.001	NA	NA	NA	NA
**GERD duration^**								
≤ 12 months	NA	NA	NA	NA	110 (70.8)	45 (29.2)	1	
> 12 months	NA	NA	NA	NA	26 (40.0)	39 (60.0)	2.06 (1.49–2.83)	< 0.001

**ASU** = Acute spontaneous urticaria

**CSU** = Chronic spontaneous urticaria

**PR°** = Prevalence ratio adjusted for all variables present in the table

* = Results from UGOS modelled vs. Urticaria or GERD

**^** = GERD duration before appearance of urticaria

**NA** = Not applicable.

Moreover, in both CSU and GERD patients the CSU and GERD overlap syndrome was significantly and independently associated with increased levels of Tot-IgE (≥ 100 IU/ml) (p = 0.002) or EBC (≥ 250 cells/mmc) (p = 0.035) without a significant interaction between these two biological parameters (Table 6).

**Table 6:** Association between chronic urticaria and GERD overlap syndrome (Chronic UGOS) with serum total IgE and eosinophils blood count in patients suffering from gastroesophageal reflux disease (GERD) [A] or chronic urticaria [B].

	CLINICAL PRESENTATION: CHRONIC UGOS [A]				CLINICAL PRESENTATION: CHRONIC UGOS [B]			
	GERD N = 136	CHRONICUGOS N = 70			CSU N = 50	CHRONIC UGOS N = 70		
	n (%)	n (%)	*Adjusted PR°* *(CI 95%)*	p value*	n (%)	n (%)	*Adjusted PR °* *(CI 95%)*	p value*
**Total IgE (IU/ml)**								
• < 100	106 (88.3)	14 (11.7)	1		38 (73,1)	14 (26,9)	1	
• ≥ 100	30 (34.1)	56 (65.1)	11.18 (2.42–51.62)	0.002	12 (17,6)	56 (82,4)	4.88 (1.12–21.29)	0.035
**EBC°° (cells/mmc)**								
• < 250	96 (92.3)	8 (7.7)	1		34 (80,9)	8 (19,1)	1	
• ≥ 250	40 (39.2)	62 (60.8)	12.95 (3.05–55.01)	0.001	16 (20,5)	62 (79,5)	6.00 (1.49–24.20)	0.012
**Total IgE & EBC**^**§**^								
- Total IgE <100 and/or EBC < 250	122 (85.9)	20 (14.1)	1		48 (70,6)	20 (29,4)	1	
- Total IgE ≥100 and EBC ≥ 250	14 (21.9)	50 (78.1)	0.22 (0.04–1.10)	0.065	2 (3,8)	50 (96,2)	0.43 (0.09–1.99)	0.277

**CSU** = Chronic spontaneous urticaria

**PR°** = Prevalence ratio adjusted for all variables present in the table

* = Results by chronic UGOS modelled vs. GERD or CSU

**EBC°°** = Eosinophils blood count

**§** = Interactions between Tot-IgE and EBC.

Finally, endoscopic and bioptic data revealed a significantly higher prevalence of NERD in patients suffering from CSU and GERD overlap syndrome (61.4%) compared to those only suffering from GERD (40.6%) as well as an increased prevalence of short BE in the former group compared to the estimated general epidemiological forecast among patients with GERD [39] (Table 7).

**Table 7:** Esophageal endoscopic findings in 205 patients suffering from gastro-esophageal reflux disease (GERD) with or without the overlap of chronic spontaneous urticaria.

	CHRONIC UGOS (*N* = 70)	GERD WITHOUT URTICARIA (*N* = 136)	*p-value*
*ENDOSCOPY/BIOPTIC DATA*	n (%)	n (%)	
• **NERD**	**43 (61.4)**	**55 (40.4)**	***0*.*004***
• **ERD Los Angeles Grade A**	**14 (20.0)**	**50 (36.8)**	***0*.*014***
• **ERD Los Angeles-Grade B**	**10 (14.3)**	**23 (16.9)**	***0*.*626***
• **ERD Los Angeles-Grade C**	**0 (0.0)**	**6 (4.4)**	***0*.*075***
• **ERD Los Angeles-Grade D**	**0 (0.0)**	**0 (0.0)**	***NC***
• **Barrett’s esophagus**	3* **(4.3)**	2* **(1.5)**	***0*.*214***

**UGOS =** Urticaria and GERD Overlap Syndrome; **Chronic UGOS** = Chronic Urticaria and GERD overlap Syndrome; **NERD =** Non-erosive reflux disease; **ERD =** Erosive reflux disease

* = Short Barrett’s esophagus cases with histological intestinal metaplasia

**NC** = Not calculable

## Discussion

To our knowledge, this is the first study evaluating the prevalence of GERD in patients with chronic and acute urticaria. Our analysis revealed that GERD was confirmed in 94% of all cases from endoscopic findings. The overall prevalence of urticaria and GERD overlap syndrome was approximately 6% and patients affected from acute or chronic variants of this syndrome presented a grade of urticaria severity that was more intense than that observed in patients suffering only from ASU or CSU respectively. Patients with a chronic urticaria overlapping a GERD were significantly more frequent than those with overlap of acute urticaria and showed Tot-IgE levels and EBCs that were more elevated than in patients without the overlapping syndrome. Finally, in cases of CSU overlapped to GERD there was a higher prevalence of NERD and, although not statistically significant, (probably because of reduced statistical power related to the small number of cases), an unusual high frequency of Barrett’s esophagus compared to that observed in patients suffering only from GERD.

The overall prevalence of urticaria was in line with that estimated in the general population [3] although the value of CSU prevalence slightly exceeded that specifically reported [2]. The overall prevalence of GICs (45%) we found in patients with CSU was greater than 26.2% and 12.8% recently reported, respectively, in American adults [27] and Turkish children [5] but was similar to that reported several years ago in European adults [40]. Though the prevalence of GERD and IBS largely vary in the general population according to different geographical location of examined population and/or to different frequency criteria of symptoms that are considered crucial for clinical diagnosis [32, 41, 42], in our sample the overall prevalence of each syndrome was similar to that reported in the European general population [32, 43, 44].

However, the observed prevalence values of these syndromes in urticaria patients were more than three-fold higher than those in patients without urticaria. Interestingly, patients with CSU had a risk of overlap with GERD that was about three-fold that in patients with ASU and, even after adjusting for differences in age, sex and HP co-infection distributions between these two groups, it was strongly associated with relatively high levels of Tot-IgE and EBC. However, although overlap of IBS and GERD as well as other functional gastrointestinal syndromes is very frequent in the general population [45–48], the overlap syndrome of CSU and GERD that we report is interesting. Clinically, this phenotype of CSU was found to be more severe and similar to what was recently reported for CSU cases with overlap of systemic complaints [27]. A dysregulated neurogenic inflammation [49], which is highlighted in GERD and in CSU by presence of relatively elevated plasma levels of neuropeptide SP [20,30,31], can be a possible pathway that explains the overlap of CSU with these gastrointestinal syndromes. To date, elevated serum levels of SP have only been reported in one study in GERD [30] while the few studies that investigated this topic in CSU-patients showed conclusions that were not completely consistent regarding possible differences with control subjects [20, 31, 50].

Although the association between mucosal neurogenic inflammation of GERD and cutaneous neurogenic inflammation of CSU as well as those between serum SP levels and both neurogenic inflammations are currently unknown, preferential activation by SP of human skin MCs via MRGPRX2 [21], and the associate over-expression of this receptor on skin MCs in CSU patients [22] may be a potential connection between mucosal neurogenic inflammation and the onset of CSU. Furthermore, the observed stepwise and parallel increase in Tot-IgE and EBC values, from the lowest in GERD-patients without urticaria to the highest in patients with overlap syndrome of CSU and GERD, suggests a progressively increasing activation of a Th2-like profile of immune-inflammatory responses because of a CSU appeared months or years after a longstanding GERD that had previously been poorly characterized as well as discontinuously managed. The increase in IgE, which is associated with the transition from a previous isolated GERD to an overlap syndrome of GERD with acute or chronic urticaria, suggests that the presence or absence of urticaria in patients with a history of GERD can play a key role in determining total serum levels of IgE. However, the specific finding of a significant increase in circulating eosinophils in patients suffering from CSU overlapping GERD compared to patients with ASU overlapping GERD or isolated CSU suggests that this phenomenon may result from a synergistic biological effect of both diseases but that it is predominantly related to the duration of the overlap syndrome.

Eosinophilic esophagitis, GERD and other chronic dysfunctional gastrointestinal diseases have recently been revisited as possible expression of atopy/food allergy [51–55]. Actually, levels of total/specific IgE as well as expression rate of allergic diseases slightly higher than in the general population have already been reported in patients with CSU [56–58]. Moreover, the skin MC_TC_ subset, the main actor of CSU history, largely depends on its multiple and not yet fully clarified functions by activation of FcεRI-IgE axis, among others also by auto-allergens [8–12, 25, 59, 60]. This is all true considering that in experimental animal models it has elegantly been demonstrated that perivascular skin MC_TC_ are able to exhibit a selective uptake of IgE by their positioning and by extending a cell process across the vessel wall to capture intraluminal IgE [61].

Unfortunately, in GERD or other chronic gastrointestinal syndromes as well as more and more in CSU where there are often altered skin responses, gastrointestinal synthesis of food-specific IgE may be difficult to be detected in the blood or skin due to their minute levels that could well be below the sensitivity threshold of diagnostic methods we currently use [62, 63].

Corroborating findings from other CSU reports [26], also in our patients, no significant blood eosinophilia was observed. However, SP has recently been shown to activate human eosinophils promoting their survival by negative modulation of apoptosis as well as exerting a prochemotactic effect on these cells [24]. Moreover, prostaglandin D2, which is a potent proinflammatory mediator mainly synthesized and released from activated mast cells, induces a strong chemotactic effect on eosinophils, basophils and Th2 lymphocytes through its DP2 receptor that is predominantly expressed in these cells [64, 65] and promotes their migration into the skin [66]. Indeed, eosinophils cumulate in skin lesions of CSU where they colocalize with MC_TC_ and induce a further activation of these cells [22, 25]. Importantly, NERD and BE, which showed a higher frequency in overlap syndrome of CSU and GERD, have recently been associated to a Th2-like profile of systemic inflammatory response with increased systemic expression of interleukin 4 and 10 [67]. With regard to CSU, it has recently been shown that the expression of Th2-initianting cytokines like IL-33 and IL-25 is increased and, among others, these cytokines co-localize with MCs in the lesional skin of patients suffering from CSU [8, 68].

On this basis, we speculate that in the presence of a longstanding GERD, the slightly elevated serum levels of SP as well as low levels of IgE initially produced in the gut could progressively activate gut MCs and the small subset of MC_TC_ in the submucosa, as well as MC_TC_ in the skin, where SP would act initially via MRGPRX2 present on intravascular processes of these cells [21, 61]. The ongoing activation of MCs that are in intimate synaptic-like contact with sensory peptidergic nerves [69–72] not only initiates a vicious circle enhancing both MC activation and neurogenic inflammation, but also may implement, through some mediators of the induced Th2-like “proinflammatory soup”, an increase in IgE production at anatomical sites originally involved as well as their increasing blood spreading with relative increase of Tot-IgE [73, 74]. Similarly, the negative modulation of SP on eosinophils apoptosis could induce a relative increase in the number of these cells in the blood but the described prochemotactic effect on these cells of same SP as well as of prostaglandin D2 could determine progressive accumulation of eosinophils in urticarial lesions so that a blood eosinophilia is never induced. In conclusion, we observed that GERD was the most frequent GIC in patients with urticaria. The prevalence of urticaria and GERD overlap syndrome was much higher in CSU-patients, among whom the overlapping syndrome was associated with more intense urticarial symptoms, higher Tot-IgE and EBC levels, and higher frequencies of NERD and BE. These results as a whole suggest that urticaria and GERD overlap syndrome is frequently a clinical chronic syndrome with a prevalent Th2-like profile.

## Supporting information

S1 Dataset Minimal anonymized dataset necessary to replicate our study findings. **Legend symbols**: **GERD** = gastroesophageal reflux disease; **EBC** = Eosinophils blood count; **Tot IgE** = serum total IgE level; **HP** = Helicobacter Pylori infection; *URT-DUR = duration of urticaria (weeks); **GERD-DUR = duration of gastroesophageal reflux disease before urticaria onset (months); **UAS7** = urticaria activity score according to UAS7; **IBS =** irritable bowel syndrome; ***Clinical diagnoses: **ASU** = acute spontaneous urticaria; **CSU** = chronic spontaneous urticaria; **acute/chronic UGOS** = acute/chronic Urticaria and GERD Overlap Syndrome; **IBD =** inflammatory bowel diseases; ****Endoscopic study: endoscopy/bioptic data of GERD according to Los Angeles Classification (L.A.)–grade A, B, C, D (L.A. A/B/C/D); **NERD** = Non erosive disease; **S-BE** = Short-Barrett’s esophagus; bioptic data of celiac disease (CD) according to the modified Marsh–Oberhuber classification–type 1–4 (Marsh 1–4).
(XLSX)
